# Immune correlates of protection following Rift Valley fever virus vaccination

**DOI:** 10.1038/s41541-022-00551-4

**Published:** 2022-10-28

**Authors:** Joshua D. Doyle, Dominique J. Barbeau, Haley N. Cartwright, Anita K. McElroy

**Affiliations:** 1 University of Pittsburgh School of Medicine, Department of Pediatrics, Division of Pediatric Infectious Disease, Pittsburgh, PA 15224 USA; 2 University of Pittsburgh, Center for Vaccine Research, Pittsburgh, PA 15261 USA; 3 UPMC Children’s Hospital, Pittsburgh, PA 15224 USA

**Keywords:** Vaccines, Infection, Virology

## Abstract

Rift Valley fever virus (RVFV) is a hemorrhagic fever virus with the potential for significant economic and public health impact. Vaccination with an attenuated strain, DelNSsRVFV, provides protection from an otherwise lethal RVFV challenge, but mechanistic determinants of protection are undefined. In this study, a murine model was used to assess the contributions of humoral and cellular immunity to DelNSsRVFV-mediated protection. Vaccinated mice depleted of T cells were protected against subsequent challenge, and passive transfer of immune serum from vaccinated animals to naïve animals was also protective, demonstrating that T cells were dispensable in the presence of humoral immunity and that humoral immunity alone was sufficient. Animals depleted of B cells and then vaccinated were protected against challenge. Total splenocytes, but not T cells alone, B cells alone, or B + T cells harvested from vaccinated animals and then transferred to naïve animals were sufficient to confer protection, suggesting that multiple cellular interactions were required for effective cellular immunity. Together, these data indicate that humoral immunity is sufficient to confer vaccine-mediated protection and suggests that cellular immunity plays a role in protection that requires the interaction of various cellular components.

## Introduction

Rift Valley fever virus (RVFV) is a mosquito-borne zoonotic virus endemic to Africa and the Arabian Peninsula. Outbreaks occur in humans, with substantial morbidity and mortality^1–4^. Rift Valley fever (RVF) is typically a self-limited febrile illness with accompanying myalgias, arthralgias, and headache. However, some patients experience severe disease including hepatitis, encephalitis, retinitis, or death. The overall mortality rate of RVF is estimated to be ~2%^5^. Due to the potential agricultural and public health impact of RVFV in non-endemic countries, RVFV is classified as a Category A overlap select agent by the Centers for Disease Control and United States Department of Agriculture^5^. There are currently no RVFV vaccines licensed for use in humans.

There have been several historical attempts to develop a vaccine against RVFV. In the 1940s, vaccination with a central nervous system (CNS)-adapted strain of RVFV provided subsequent protection against challenge with wild-type (WT) RVFV, and passive transfer of serum from immunized lambs protected naïve mice^6^. Variants of this live attenuated strain were used in livestock vaccination campaigns throughout the 1950s^7^. In the 1960s, a formalin-inactivated RVFV vaccine elicited robust neutralizing antibody titers and protection from challenge in mice and non-human primates; this vaccine was given to laboratory workers at risk of occupational RVFV exposure^8–10^. Cell-adapted, formalin-inactivated RVFV vaccines were subsequently immunogenic in sheep^11^ and saw agricultural use. More recently, a strain of RVFV generated by reverse genetics and lacking the virulence factors NSs and NSm has demonstrated immunogenicity and safety in a variety of animal model systems, including rodents, sheep, and non-human primates^12–14^. Multiple other RVFV vaccines have undergone preclinical studies, including a chimpanzee adenovirus vectored vaccine^15^, a 4-segmented RVFV vaccine^16^, and a capripoxvirus vectored vaccine^17^.

Despite the long history of RVFV vaccine development efforts, the mechanistic correlates of protection following RVFV vaccination remain poorly understood. A number of studies have demonstrated that neutralizing antibody titers correlate with protection against WT RVFV infection^6,8,11^. Accordingly, passively transferred immune sera can protect naïve animals against challenge^6,18,19^. The role of cell-mediated immunity in controlling and modulating RVFV infection is less clear. Clearance of an attenuated vaccine strain of RVFV, DelNSsRVFV, is dependent on B cells and CD4+ T cells, and mice depleted of CD4+ T cells have an increased incidence of encephalitis compared with mock-depleted animals^20^. Additionally, 90% of mice with genetic B-cell ablation (µMT mice) were clinically well following DelNSsRVFV vaccination, but succumbed to disease following WT RVFV challenge^20^; however, the fact that µMT mice also have aberrant T-cell development complicates interpretation. Subsequent work suggested that protection from encephalitis involves a coordinated response involving T cells and monocytes^21^ and that this response is likely dependent on CD40/CD40L interactions as well as T follicular helper cells^22^. Field trials of formalin-inactivated vaccines in the 1970s reported robust resistance to natural RVFV infection in vaccinated sheep and cattle despite low levels of neutralizing antibody titers, suggesting a role for cellular immunity in vaccine-mediated protection^11,23,24^. Additionally, T-cell responses were detectable in human recipients of a formalin-inactivated vaccine decades after immunization^25^.

In this study, the mechanistic immune correlates of protection following DelNSsRVFV vaccination of immunocompetent mice were investigated. This strain was selected (as opposed to DelNSsDelNSmRVFV) since much of our previous work has been done using DelNSsRVFV; additionally, DelNSmRVFV retains pathogenicity in the mouse model, suggesting that NSs rather than NSm is the major virulence factor in mice. DelNSsRVFV vaccination-derived humoral immunity of adequate titer was able to provide complete protection against WT Rift Valley fever virus challenge; combined cellular immunity could also provide protection, but a role for sub-therapeutic antibodies in this context could not be ruled out.

## Results

### T cells were not required for protection against lethal challenge following vaccination with DelNSsRVFV

**Fig. 1:**
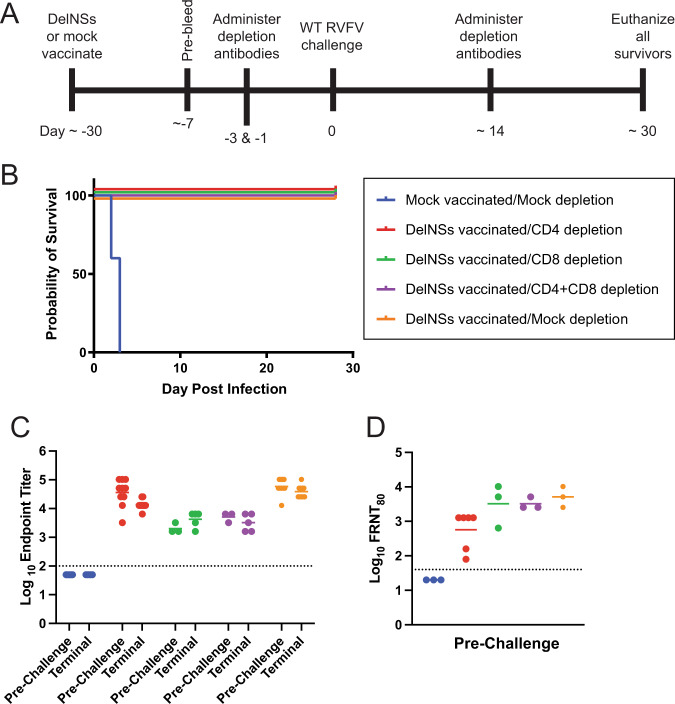
RVFV-specific memory T cells are dispensable for survival in the presence of pre-existing RVFV-specific antibodies. Mice (*n* = 5–10 per experimental group) were vaccinated with 2 × 10^5^ TCID_50_ of DelNSsRVFV or mock-vaccinated then depleted of CD4+, CD8+, CD4+/CD8+ cells or mock-depleted, then challenged with 2 × 10^5^ TCID_50_ of WT RVFV (**A**) and monitored for survival (**B**). Endpoint titers of RVFV-specific antibodies by ELISA (**C**) or neutralizing antibody titers (**D**) were measured prior to and/or after WT challenge. The dotted line is the LOD (100 for ELISA, 40 for FRNT_80_). Negative values were plotted at 50 and 20, respectively. The geometric mean titer is noted for each group.

Mice were vaccinated with DelNSsRVFV (2 × 10^5^ TCID_50_) or mock-vaccinated (Fig. 1A). Mice were then mock-depleted or depleted of CD4+ cells, CD8+ cells, or CD4+ and CD8+ cells prior to challenge with 2 × 10^5^ TCID_50_ WT RVFV. Mice underwent repeat depletion ~2 weeks after the challenge, and surviving mice were sacrificed approximately one month after WT RVFV challenge.

Mock-vaccinated mice uniformly succumbed following the challenge with WT RVFV (Fig. 1B). By contrast, all mice vaccinated with DelNSsRVFV were protected from the challenge with WT RVFV, regardless of lymphocyte depletion. Successful depletion of target lymphocyte populations was confirmed by flow cytometry (Supplementary Fig. 1).

Total anti-RVFV antibody production was assessed pre-challenge and at the time of euthanasia in all mice (Fig. 1C). Mice vaccinated with DelNSsRVFV produced robust RVFV-specific antibodies (geometric mean endpoint titer (GMT) > 2000) prior to challenge with RVFV, and had similar antibody titers following challenge regardless of lymphocyte depletion. The limit of detection (LOD) of this assay is 100, and samples testing below the LOD were assigned a value of 50.

Neutralizing antibody titers were assessed following vaccination but prior to WT challenge via an 80% focus reduction neutralization assay (FRNT_80_). All vaccinated groups had FRNT_80_ GMT > 500 prior to lymphocyte depletion. The LOD of this assay was 40, and samples testing below the LOD were assigned a value of 20. These data suggest that protection following vaccination is not dependent on T lymphocytes when virus-specific antibodies are present.

### Vaccinated mice were protected against WT RVFV challenge despite B-cell depletion

**Fig. 2:**
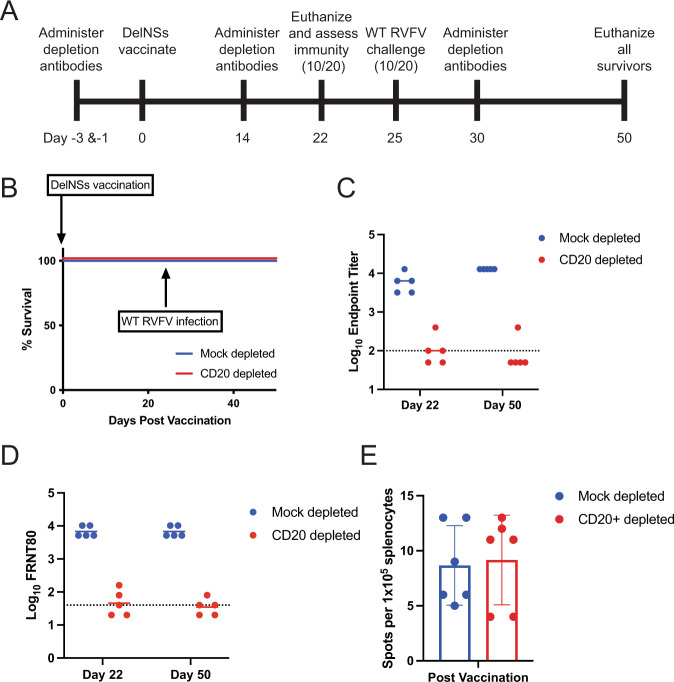
RVFV-specific antibodies are largely dispensable for survival in the presence of pre-existing RVFV-specific T cells. Mice (*n* = 10 per experimental group) were depleted of CD20+ lymphocytes or mock-depleted, then vaccinated with 2 × 10^5^ TCID_50_ DelNSsRVFV. Half were euthanized for immune assessment, and the other half were challenged with 2 × 10^5^ TCID_50_ of WT RVFV (**A**). Animals were monitored for survival (**B**). Endpoint titers of RVFV-specific antibodies by ELISA (**C**) or neutralizing antibody titers (**D**) were measured 22 days after vaccination/prior to WT challenge, and again at the end of the experiment. The dotted line is the LOD (100 for ELISA, 40 for FRNT_80_). Negative values were plotted at 50 and 20, respectively. The geometric mean titer is noted for each group. The number of RVFV-specific T cells in total splenocytes was determined by IFN-γ ELISPOT assay, expressed as the number of spots per 1 × 10^5^ splenocytes (*n* = 3, in duplicate) (**E**). Mean and standard deviation are noted.

Mice were depleted of CD20+ B cells or mock-depleted prior to vaccination with 2 × 10^5^ TCID_50_ DelNSsRVFV (Fig. 2A). They were subsequently monitored closely, although none developed signs of disease. On day 22 following vaccination, half were euthanized for immune assessment, and on day 25 the remainder were challenged with 2 × 10^5^ TCID_50_ of WT RVFV. Depletion antibodies were re-administered approximately every 2 weeks to maintain depletion. All surviving animals were euthanized on day 50 following vaccination. Depletion of B cells was confirmed via flow cytometry (Supplementary Fig. 2). All vaccinated mice survived the challenge with WT RVFV, regardless of whether they underwent B-cell depletion or were mock-depleted (Fig. 2B).

Mice depleted of CD20+ cells had substantially lower titers of RVFV-specific antibodies by ELISA (Fig. 2C) and FRNT_80_ (Fig. 2D) than those that were mock-depleted at both 22 and 50 days following vaccination. ELISA GMT in mock-depleted mice were 5571 at day 22 and 12,800 at day 50, while in CD20-depleted mice, they were 100 at day 22 and 76 at day 50. Of note, 3/5 mice euthanized 22 days following vaccination, and 1/5 mice that survived following WT RVFV challenge had RVFV antibodies at or above the limit of detection, suggesting that vaccination may result in a low level of antibody production even in CD20-depleted mice. FRNT_80_ GMT in mock-depleted mice were 6756 at both day 22 and day 50, while in CD20-depleted mice, they were 46 at day 22 and 35 at day 50. Again, some animals had detectable albeit low FRNT_80_ levels, so CD20 depletion did not eliminate all antibody production.

The number of splenocytes secreting interferon-gamma in response to RVFV peptide stimulation was quantified via enzyme-linked immunospot (ELISPOT) (Fig. 2E). Mice depleted of CD20+ cells had similar numbers of RVFV-specific splenocytes following vaccination as mock-depleted mice, indicating that the cellular response to vaccination was similar between these two groups. The survival of mice after RVFV challenge was not significantly affected by B-cell depletion, suggesting that the cellular immune response is sufficient to provide protection against RVFV disease post-vaccination, although a minor contribution from very low levels of virus-specific antibodies cannot be excluded.

### Passive transfer of RVFV-specific antibodies was sufficient to protect against lethal RVFV

**Fig. 3:**
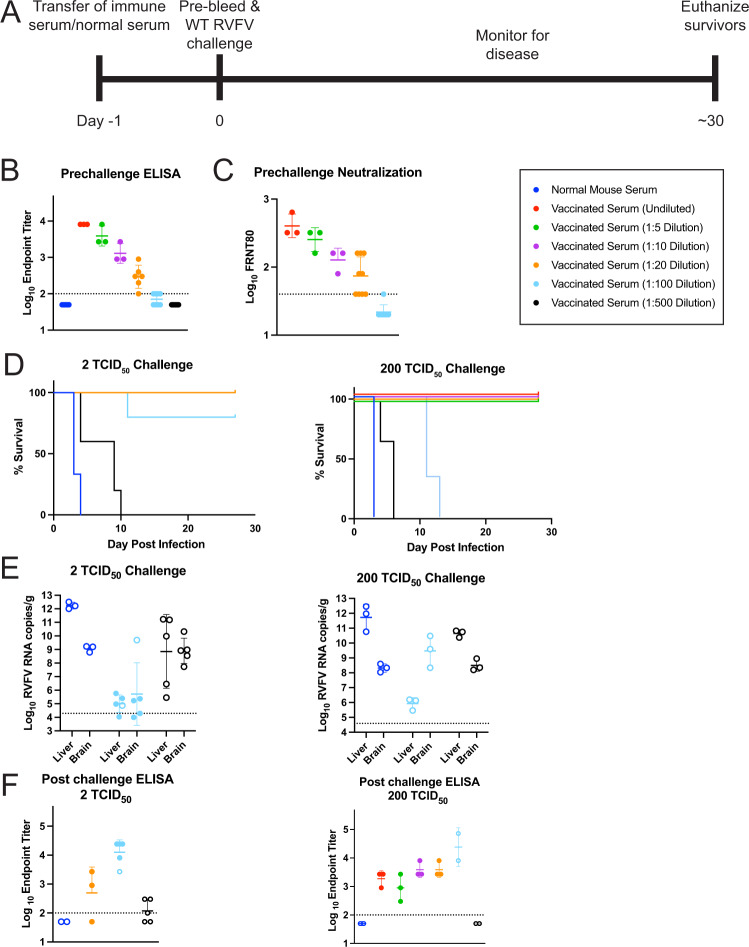
Passive transfer of immune serum can protect from lethal RVFV. Naïve mice (*n* = 3–5 per experimental group) were inoculated with 200 uL of serum from vaccinated mice, either undiluted or diluted 1:5, 1:10, 1:20, 1:100, or 1:500. Control mice were inoculated with normal mouse serum. All mice were bled then challenged with WT RVFV. **A** Endpoint titers of RVFV-specific antibodies were determined via ELISA (**B**) and FRNT (**C**) 1 day prior to challenge. Mice were then challenged with 2 or 200 TCID_50_ of WT RVFV and monitored for survival (**D**). Terminal viral RNA load (**E**) and ELISA titer (**F**) were also measured. Open circles in E&F represent animals that died or required euthanasia while closed circles represent animals that survived; the dotted line is the LOD of the assay. For ELISA and FRNT_80_ graphs the dotted line is the LOD (100 for ELISA, 40 for FRNT_80_). Negative values were plotted at 50 and 20, respectively. Geometric means and geometric standard deviation are noted.

Naïve mice were inoculated with 200 µL of serum from DelNSsRVFV vaccinated mice at various dilutions, then challenged with 2 or 200 TCID_50_ WT RVFV (Fig. 3A). Control mice were inoculated with serum from unimmunized (naïve) mice prior to challenge. Endpoint titers of RVFV-specific antibodies were assessed by ELISA (Fig. 3B), and for mice with detectable ELISA titers, FRNT (Fig. 3C) assays were also performed, prior to the challenge with WT virus. Mice receiving control sera had no detectable antibodies, and mice receiving immune sera had pre-challenge titers that correlated with the dilutions received. Neutralizing antibodies were detected in only 1 out of 5 mice receiving immune serum diluted 1:100 (Fig. 3C). Mice receiving control serum succumbed to infection 3–4 days after the challenge, and mice receiving immune serum diluted 1:500 all succumbed at both challenge doses but in a delayed manner. Mice receiving immune serum diluted 1:100 all succumbed to late-onset encephalitis at a challenge dose of 200 TCID_50_, but most survived at the 2 TCID_50_ dose. Mice receiving undiluted immune serum or serum diluted 1:5, 1:10, or 1:20 all survived regardless of challenge dose (Fig. 3D). Viral RNA loads were highest in the liver in mice that succumbed (open circles) in the first-week post-infection and highest in the brain in mice that succumbed in the second-week post-infection (Fig. 3E). Surviving mice (filled circles) had viral RNA loads near the LOD of the assay. Post-challenge ELISA titers demonstrated that mice receiving undiluted immune serum or serum diluted 1:5 had decreased titers at the time of euthanasia; however, those receiving lower concentrations of serum (1:10, 1:20, and 1:100) showed increased antibody titers at the time of euthanasia, suggesting a de novo immune response to challenge in mice receiving lower doses of antibodies (Fig. 3F). Notably, in the mice receiving immune serum diluted 1:100 and challenged with 200 TCID_50_ of WT RVFV, neither the low level of administered antibodies nor the de novo immune response was able to provide protection from RVFV encephalitis.

Passive transfer of sera from immunized animals to naïve hosts was sufficient to protect 100% of mice from lethal challenge at the doses evaluated when an ELISA GMT of 294 or above or an FRNT_80_ GMT of 89 or above was achieved.

### Adoptive immune splenocyte but not T-cell or B-cell transfer protected against RVFV challenge

**Fig. 4:**
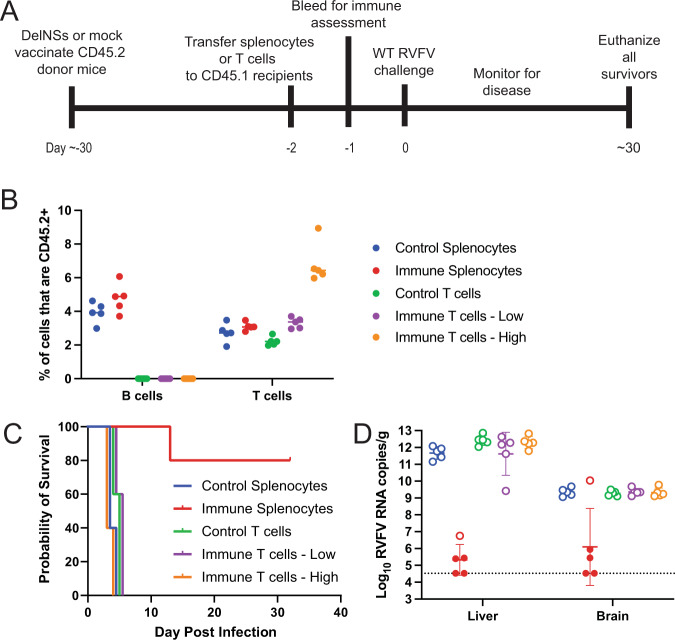
Adoptive transfer of splenocytes from vaccinated animals provides protection from WT challenge. Naïve CD45.2 mice (*n* = 5 per experimental group) were vaccinated with 2 × 10^5^ TCID_50_ of DelNSsRVFV or mock-vaccinated. Control or immune total splenocytes or T cells were harvested and transferred to naïve CD45.1 recipient mice (**A**). Recipient mice received immune splenocytes, control splenocytes, control T cells, or immune T cells at low (1 × 10^7^) or high (2.6 × 10^7^) concentrations. The number of donor-derived (CD45.2) B cells and T cells present in the blood of recipient animals one day after transfer was determined by flow cytometry (**B**). Mean values are noted. Mice were subsequently challenged with 2 TCID_50_ WT RVFV and monitored for survival (**C**). Viral RNA loads were determined at the time of euthanasia (**D**). Open circles represent animals that died or required euthanasia while closed circles represent animals that survived. The dotted line is the LOD of the assay. Geometric means and geometric standard deviation are noted.

As shown above, vaccination protected mice against virulent challenge even after the depletion of CD20+ B cells, suggesting that cellular immunity might be sufficient to provide protection. To test this, naïve CD45.2 mice were DelNSsRVFV or mock-vaccinated. Approximately 1 month later, total splenocytes, T cells, or B cells were purified from vaccinated mice and transferred to naïve CD45.1 mice prior to WT RVFV challenge. Using CD45.2 donors and CD45.1 recipients allowed differentiation between donor- and recipient-derived lymphocytes (Supplementary Fig. 3). Mice were bled prior to challenge to quantitate transferred cell populations by flow cytometry (Supplementary Fig. 4B, Fig. 4B). As expected, animals receiving total splenocytes had detectable populations of donor B cells and T cells, and no notable differences were observed between those receiving control and immune splenocytes. None of the mice receiving T cell infusions had detectable populations of donor-derived B cells, and those in the group receiving the ‘High’ concentration of T cells accordingly had a higher percentage of donor-derived T cells.

Recipient mice were then inoculated with 2 × 10^5^ TCID_50_ (Supplementary Fig. 4) or 2 TCID_50_ WT RVFV (Fig. 4) and monitored for disease. Mice receiving any cell preparation from mock-vaccinated animals uniformly succumbed to WT RVFV challenge within 3–4 days, but 80% of those receiving 1–5 × 10^7^ total donor splenocytes from animals immunized with DelNSsRVFV survived to 25 days post-challenge without evidence of disease regardless of challenge dose (Supplementary Fig. 4C and Fig. 4C). Animals receiving 1 × 10^7^ total T cells or B cells from immunized donors uniformly succumbed to the disease at a high challenge dose (Supplementary Fig. 4C). We hypothesized that at the high challenge dose the number of T cells transferred was inadequate to confer protection at the ‘Low’ concentration of 1 × 10^7^ T cells per recipient, which contained approximately 4800 RVFV-specific T cells per animal by ELISPOT. Accordingly, this experiment was repeated with a ‘High’ concentration of 2.6 × 10^7^ T cells, which contained ~28,000 RVFV-specific T cells per animal by ELISPOT; however, these animals also succumbed to a 2 TCID_50_ WT challenge (Fig. 4C). By comparison, transferred total splenocytes were calculated to contain ~20,000 RVFV-specific cells per animal by ELISPOT, so despite administration of an equivalent number of RVFV-specific T cells, T cells given alone were not able to protect. Viral RNA loads were measured in all mice at the time of euthanasia. Mice that died (open circles) in the first week all had the highest viral RNA loads in the liver, while the one mouse that died in the immune splenocyte group died later with high viral RNA loads in the brain. All surviving mice (solid circles) had viral RNA loads near the LOD of the assay (Fig. 4D).

### Adoptive transfer of T cells and B cells or splenocytes depleted of B cells did not confer protection

**Fig. 5:**
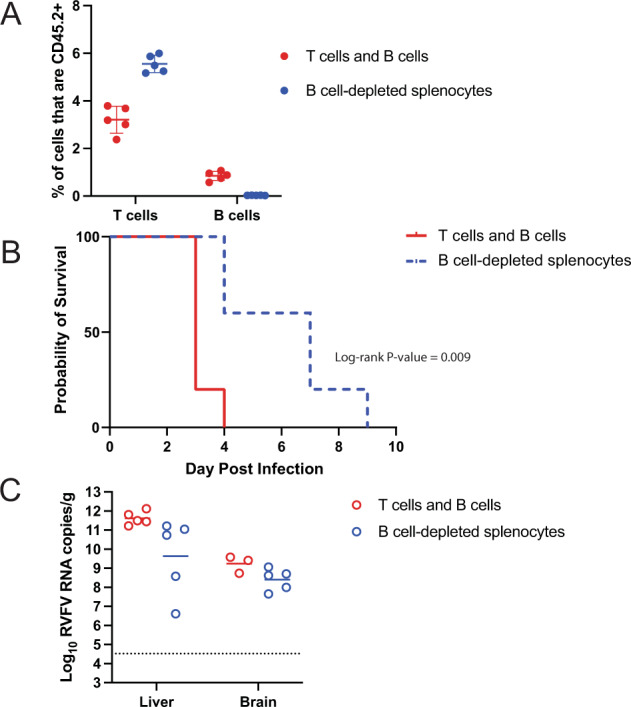
Neither adoptive transfer of B and T cells or B cell-depleted splenocytes was sufficient to protect from WT challenge. Naïve CD45.2 mice (*n* = 5 per experimental group) were vaccinated with 2 × 10^5^ TCID_50_ of DelNSsRVFV. On day 30 post-vaccination, total splenocytes were harvested and subjected to either negative selection for B and T cells or positive selection to deplete B cells. Naïve recipient CD45.1 mice were given either a 1:1 mixture of immune B and T cells or B cell-depleted immune splenocytes. The number of donor-derived (CD45.2) B cells and T cells present in the blood of recipient animals 1 day after transfer was determined by flow cytometry (**A**). Mean and standard deviation are noted. Mice were challenged with 2 TCID_50_ WT RVFV, and then monitored for survival (**B**). Significance was assessed by log-rank test. Viral RNA loads were determined at the time of euthanasia (**C**). The dotted line is the LOD of the assay. Geometric mean titer and geometric standard deviation are noted.

Adoptive transfer of total splenocytes from vaccinated animals was sufficient to confer protection against WT challenge in naïve recipients; however, T cells alone were insufficient for protection, even when equivalent numbers of RVFV-specific T cells were administered. From this finding, we hypothesized that additional cellular interactions may be required between RVFV-specific T cells and other populations of cells present in total splenocytes. To test this, we vaccinated naïve CD45.2 mice, harvested total splenocytes, then independently selected populations of T cells and B cells using negative selection. Separately, an additional group of naïve CD45.2 mice was vaccinated, and total splenocytes were harvested, then depleted of B cells using positive selection. Naive CD45.1 recipient mice were inoculated with either a mixture of purified T cells and B cells (2 × 10^7^ total cells/animal in a 1:1 ratio; 11,000 RVFV-specific T cells by ELISPOT) or B cell-depleted splenocytes (2 × 10^7^ total cells/animal; 22,000 RVFV-specific T cells by ELISPOT), then challenged with 2 TCID_50_ WT RVFV (Fig. 5). Assessment of donor cell frequency in recipient mice demonstrated the expected ratios of donor B and T cells in recipients (Fig. 5A). Mice receiving a mixture of T cells and B cells succumbed to infection in 3–4 days, similar to those receiving T cells alone. Interestingly, mice receiving B cell-depleted splenocytes had a longer median time to death (7 days), although all eventually succumbed (Fig. 5B). Viral RNA loads at the time of euthanasia were highest in the liver (Fig. 5C).

## Discussion

Humoral immunity has long been considered an important correlate of protection for vaccines against a wide variety of viruses, including RVFV^10,24,26,27^. It has also been established that cell-mediated immunity is critical in modulating the pattern and outcome of RVFV response to infection^20,21^. Moreover, vaccination with a DNA construct encoding the RVFV nucleoprotein (N) afforded at least partial protection, presumably mediated by cellular immunity, as N is not a surface protein and would thus not be expected to elicit a protective neutralizing antibody response^28^. However, the nature of the cellular responses induced by vaccination with DelNSsRVFV, and the contribution they make to vaccine-mediated protection, have not been well defined.

In this study, we show that vaccination with DelNSsRVFV protected mice from WT challenge even in the absence of T cells, suggesting that cellular immunity was not required for vaccine-mediated protection. Animals depleted of B cells and then vaccinated were also protected from challenge, suggesting that cellular immunity alone is protective, although a low level of RVFV-specific antibody was detectable in some animals despite B cell depletion. Transfer of immune serum from vaccinated animals to naïve hosts also provided protection against challenge at the evaluated doses so long as an ELISA GMT of >294 was administered. Interestingly, animals receiving high concentrations of transferred antibodies had diminished titers at the time of euthanasia, several weeks after WT challenge, but animals receiving lower concentrations of transferred antibodies had increased titers at the time of euthanasia. This finding suggests that high concentrations of a transferred antibody provided sterilizing immunity, abrogating WT viral replication in naïve hosts, and these transferred antibodies subsequently waned from recipient animals by the end of the experiment. However, lower levels of transferred antibody may be insufficient to prevent early rounds of WT viral replication, but provide sufficient protection to prevent death. In this situation, the naïve host adaptive response to WT challenge may stimulate new antibody production, resulting in the rise in antibody titer seen at the time of euthanasia, and indicating that even at concentrations insufficient for complete neutralization, transferred antibody is able to coordinate with a naïve immune system to control RVFV infection. Lastly, we found that the adoptive transfer of immune splenocytes, but not immune T cells or B cells, was sufficient to protect most naïve recipient mice. Mice receiving immune splenocytes initially lost weight following WT challenge; however, they regained their starting weight by the end of the experiment, suggesting that the presence of an RVFV-specific cellular immune response at the time of challenge is able to limit and ultimately eliminate the infection.

The precise roles of T cells, B cells, and other cellular immune mediators in RVFV vaccine-mediated protection is more complex. Work in other viral systems has established a role for immune T cells in mediating viral clearance and protecting against subsequent challenges. For instance, CD8+ T cells are critical in the control of lymphocytic choriomeningitis virus (LCMV), and adoptive transfer of CD8+ T cells resulted in reduced viral titer in infected animals^29^. However, B cells and CD4+ T cells were required for complete clearance of LCMV^30–32^. Similarly, a mouse model of Ebola virus infection demonstrated a critical role for CD8+ T cells in providing protection against lethal viral challenge^33^; transfer of immune CD8+ T cells alone was sufficient to afford protection against lethal challenge in naïve animals^34^. Studies of Lassa fever virus have shown that adoptive transfer of immune splenocytes, but not splenocytes depleted of total T cells^35^ or CD8+ T cells^36^, conferred protection from subsequent challenge. In this context, it is not immediately clear why the transfer of immune splenocytes conferred protection against RVFV, whereas the transfer of immune T cells alone, B cells alone, T cells and B cells together, or splenocytes depleted of B cells all did not. Adoptive transfer of B cell-depleted splenocytes did not confer full protection against challenge, although the median time to death was intriguingly increased compared with naïve mice. It is possible that multifactorial signaling events are required between T cells, B cells, and non-B/non-T splenocytes to confer full protection. Another potential explanation is that in the setting of intact cellular immunity, an extremely small quantity of antibodies may be sufficient to augment a protective immune response. Given the rapid lethality of RVFV infection in this murine model, it is plausible that a small number of antibodies may alter the kinetics of infection sufficiently to permit clearance by an adaptive T cell response. A limitation of this model is that these results may not be perfectly generalizable to human RVFV infections, given the different time courses and patterns of disease observed in human patients.

Taken together, these findings provide insight into the mechanistic correlates of protection conferred by vaccination against RVFV. We have shown that vaccination with DelNSsRVFV induces both cellular and humoral responses, and that vaccination still confers protection after substantial ablation of either. The precise interplay between antibodies and cellular immune components required for protection requires further study.

## Methods

### Ethics statement

This study complied with institutional guidelines, the US Department of Agriculture Animal Welfare Act, and the National Institutes of Health Guidelines for the humane use of laboratory animals. All procedures were approved by the University of Pittsburgh Institutional Animal Care and Use Committee (Protocols 17080998, 19044158, and 22030821).

### Mice, viruses, and biosafety

All work with infectious RVFV was completed in a biosafety level (BSL)-3 laboratory. Female 6–10-week-old WT C57BL/6 J and CD45.1 C57BL/6 mice were obtained from Jackson Laboratories and housed within ABSL-3 laboratories in microisolator pans in HEPA filtration racks, following standard barrier techniques. In all experiments, mice were evaluated for clinical signs of disease at least once daily and were euthanized according to a predetermined clinical illness scoring algorithm^37^. At the time of euthanasia, mice were anesthetized with isoflurane and terminally bled. Collected specimens included liver, spleen, brain, popliteal lymph node, and/or whole blood. Splenocytes and other tissues were processed using manual disruption^38^.

Stocks of recombinant WT RVFV (strain ZH501) and RVFV lacking NSs (DelNSsRVFV) were produced using reverse genetics and grown to passage 2^12,39^. Titers of all viral stocks were determined as the 50% tissue culture infective dose (TCID_50_) on Vero E6 cells and visualized by indirect fluorescent-antibody assay (IFA) using a monoclonal mouse IgG1 anti-RVFV N primary antibody (custom, Genscript). Virus sequence identity was verified prior to use.

### Lymphocyte depletions

Mice were depleted of B cells, CD4+ T cells, or CD8+ T cells using monoclonal anti-CD20 (SA271G2), anti-CD4 (GK 1.5), or anti-CD8 (YTS 169.4) antibodies, respectively, or were mock-depleted using an isotype control antibody (LTF2) (all obtained from Bio X Cell except SA271G2, obtained from BioLegend (BL)). For CD4+ , CD8+ , and CD4+/CD8+ depletions, antibodies were diluted in sterile phosphate-buffered saline (PBS), and 300 µg was administered intraperitoneally (IP) to each mouse on days 3 and 1 prior to RVFV challenge and once following RVFV challenge. For CD20 depletion, antibodies were diluted in PBS, and 200 µg was administered to each mouse IP on days 3 and 1 prior to vaccination with DelNSsRVFV, and two additional times (on days 14 and 30) following vaccination. Depletion efficiency was determined by flow cytometry of indicated tissues at euthanasia. Tissues were prepared using manual disruption^18^, and flow cytometry was performed as described below.

### Serology

Serum was collected and used for an indirect RVFV whole lysate ELISA^40^. The endpoint ELISA titer for each mouse was defined as the highest dilution of serum that resulted in an OD value at least three standard deviations above the average of a negative control mouse.

### Lymphocyte purification and adoptive transfer

Donor CD45.2 C57BL/6 mice were immunized with 2 × 10^5^ TCID_50_ of DelNSsRVFV via footpad (FP) injection or mock/unimmunized. On day ~30 after vaccination, donor mice were euthanized, spleens were harvested, and splenocytes were generated by mechanical disruption^38^. Total splenocytes were subjected to negative selection for T cells using an EasySep Mouse T Cell Isolation Kit, negative selection for B cells using an EasySep Mouse B Cell Isolation Kit, or positive selection to remove CD19+ B cells using an EasySep Mouse CD19 Positive Selection Kit per manufacturer’s instructions (Stemcell Technologies). Naïve CD45.1 C57BL/6 recipient mice received transfusions of total splenocytes, purified T cells, purified B cells, purified T and B cells, or splenocytes depleted of CD19+ B cells via retroorbital injection and were subsequently challenged with 2 or 2 × 10^5^ TCID_50_ WT RVFV via FP injection.

### Focus reduction neutralization assay

Mouse serum was serially diluted in duplicate and incubated with 200 foci-forming units of DelNSsDelNSmRVFV using standard methods^25^. Percent neutralization was calculated by comparing sample wells to wells containing viruses but no serum. The dilution of serum at which 80% of foci were neutralized is reported as FRNT_80_.

### T-cell ELISPOTS

Splenocytes were incubated with a peptide pool generated from RVFV N, Gc, and Gn structural proteins that encompass the immunodominant epitopes identified in C57BL/6 mice and assayed using an IFN-γ kit mouse ELISPOT kit (Mabtech Inc.) following manufacturer’s instructions^22^. Spots were counted on a CTL Immunospot reader.

### RNA extraction and RVFV qRT-PCR

Tissue samples were placed in sterile PBS with antimycotic/antibiotic (Thermo Fisher) and homogenized. Tissue homogenates or serum were mixed with TRIzol Reagent (Ambion), and RNA was extracted using Direct-zol RNA kit (Zymo Research) following the manufacturer’s directions. Tissue and serum RNA were assessed by RVFV qRT-PCR^37^. Reactions were run on an CFX96 (BioRad).

### Flow cytometry

Cells were washed in PBS, then incubated in live/dead near IR (Thermo Fisher) at 1:500 for 10 min. Following a wash in flow buffer (PBS with 2% FBS) cells were stained for 30 min using various combinations of the following antibodies: FITC CD45.1 (A20, Beckton Dickenson (BD553775, 1:100)), APC CD45.2 (104, BD558702, 1:100), PE CD19 (6D5, BL115507, 1:100), FITC CD11b (M1/70, BL101205, 1:200), APC F4/80 (BM8, BL123115, 1:100), BV421 CD40 (3/23, BD562846, 1:100), BUV395 CD4 (RM4-5, BD740208, 1:100), PECy7 CD8 (53-6.7,BL552877, 1:100), BV510 CD3 (17A2, BL100234, 1:100), AF488 CD3 (17A2, BL 100212, 1:100), APC CD45 (30-F11, BD 559864, 1:100), APC CD8 (53-6.7, BD553035, 1:100), or PCP-Cy5.5 CD4 (RM4-4, BL116011, 1:100). Cells were washed twice in flow buffer, then fixed in BD Fix/Perm prior to acquisition on an LSRII. Data were analyzed using FlowJo.

### Passive transfer

Naïve C57BL/6 recipient mice received 200 µL of normal mouse serum or immune mouse serum, either undiluted or diluted 1:5, 1:10, 1:20, 1:100, or 1:500 via IP injection. The next day, mice were bled and challenged with 2 or 200 TCID_50_ of WT RVFV via FP injection, then monitored for survival.

### Data handling and statistical analysis

All graphs were generated, and statistical analyses were performed using GraphPad Prism. Where applicable, the statistical test applied is noted in the figure legend.

### Reporting summary

Further information on research design is available in the Nature Research Reporting Summary linked to this article.

## Supplementary information

Supplemental Material

REPORTING SUMMARY

## Data Availability

The data that support the findings of this study and any unique reagents are available from the corresponding author upon reasonable request.

## References

[1] Mundel B, Gear J (1951). Rift valley fever; I. The occurrence of human cases in Johannesburg. S Afr. Med. J..

[2] Gear J (1955). Rift valley fever in South Africa; a study of the 1953 outbreak in the Orange Free State, with special reference to the vectors and possible reservoir hosts. S Afr. Med. J..

[3] Hoogstraal H, Meegan JM, Khalil GM, Adham FK (1979). The Rift Valley fever epizootic in Egypt 1977-78. 2. Ecological and entomological studies. Trans. R. Soc. Trop. Med. Hyg..

[4] Meegan JM (1979). The Rift Valley fever epizootic in Egypt 1977-78. 1. Description of the epizzotic and virological studies. Trans. R. Soc. Trop. Med. Hyg..

[5] Bird BH, Ksiazek TG, Nichol ST, Maclachlan NJ (2009). Rift Valley fever virus. J. Am. Vet. Med. Assoc..

[6] Smithburn KC (1949). Rift Valley fever; the neurotropic adaptation of the virus and the experimental use of this modified virus as a vaccine. Br. J. Exp. Pathol..

[7] Assaad F (1983). The use of veterinary vaccines for prevention and control of Rift Valley fever: memorandum from a WHO/FAO meeting. Bull. World Health Organ.

[8] Randall R, Binn LN, Harrison VR (1964). Immunization against rift valley fever virus. studies on the immunogenicity of lyophilized formalin-inactivated vaccine. J. Immunol..

[9] Binn LN, Randall R, Harrison VR, Gibbs CJ, Aulisio CG (1963). Immunization against Rift Valley fever. The development of vaccines from nonprimate cell cultures and chick embryos. Am. J. Hyg..

[10] Randall R, Gibbs CJ, Aulisio CG, Binn LN, Harrison VR (1962). The development of a formalin-killed Rift Valley fever virus vaccine for use in man. J. Immunol..

[11] Barnard BJ, Botha MJ (1977). An inactivated rift valley fever vaccine. J. S Afr. Vet. Assoc..

[12] Bird BH (2008). Rift valley fever virus lacking the NSs and NSm genes is highly attenuated, confers protective immunity from virulent virus challenge, and allows for differential identification of infected and vaccinated animals. J. Virol..

[13] Smith DR (2018). Attenuation and efficacy of live-attenuated Rift Valley fever virus vaccine candidates in non-human primates. PLoS Negl. Trop. Dis..

[14] Bird BH (2011). Rift Valley fever virus vaccine lacking the NSs and NSm genes is safe, nonteratogenic, and confers protection from viremia, pyrexia, and abortion following challenge in adult and pregnant sheep. J. Virol..

[15] Warimwe GM (2016). Chimpanzee adenovirus vaccine provides multispecies protection against rift valley fever. Sci. Rep..

[16] Wichgers Schreur PJ (2022). Safety and immunogenicity of four-segmented Rift Valley fever virus in the common marmoset. npj Vaccines.

[17] Wallace DB (2020). Protection of cattle elicited using a bivalent lumpy skin disease virus-vectored recombinant rift valley fever vaccine. Front. Vet. Sci..

[18] Niklasson BS, Meadors GF, Peters CJ (1984). Active and passive immunization against Rift Valley fever virus infection in Syrian hamsters. Acta. Pathol. Microbiol. Immunol. Scand. C..

[19] Peters CJ (1988). Experimental Rift Valley fever in rhesus macaques. Arch. Virol..

[20] Dodd KA, McElroy AK, Jones MEB, Nichol ST, Spiropoulou CF (2013). Rift Valley fever virus clearance and protection from neurologic disease are dependent on CD4+ T cell and virus-specific antibody responses. J. Virol..

[21] Harmon, J. R. et al. CD4 T cells, CD8 T cells, and monocytes coordinate to prevent rift valley fever virus encephalitis. *J. Virol.* **92**, e01270–18 (2018). 10.1128/JVI.01270-18 PMC6258944 30258000

[22] Barbeau DJ (2021). Identification and characterization of rift valley fever virus-specific T cells reveals a dependence on CD40/CD40L interactions for prevention of encephalitis. J. Virol..

[23] Pini A, Lund LJ, Davies FG (1973). Fluorescent and neutralizing antibody response to infection by Rift Valley fever virus. J. S Afr. Vet. Assoc..

[24] Barnard BJ (1979). Rift Valley fever vaccine–antibody and immune response in cattle to a live and an inactivated vaccine. J. S Afr. Vet. Assoc..

[25] Harmon JR, Barbeau DJ, Nichol ST, Spiropoulou CF, McElroy AK (2020). Rift Valley fever virus vaccination induces long-lived, antigen-specific human T cell responses. npj Vaccines.

[26] Niklasson B, Peters CJ, Bengtsson E, Norrby E (1985). Rift Valley fever virus vaccine trial: study of neutralizing antibody response in humans. Vaccine.

[27] Morrill JC (1991). Further evaluation of a mutagen-attenuated Rift Valley fever vaccine in sheep. Vaccine.

[28] Lorenzo G, Martín-Folgar R, Hevia E, Boshra H, Brun A (2010). Protection against lethal Rift Valley fever virus (RVFV) infection in transgenic IFNAR(-/-) mice induced by different DNA vaccination regimens. Vaccine.

[29] McIntyre KW, Bukowski JF, Welsh RM (1985). Exquisite specificity of adoptive immunization in arenavirus-infected mice. Antivir. Res..

[30] Planz O (1997). A critical role for neutralizing-antibody-producing B cells, CD4(+) T cells, and interferons in persistent and acute infections of mice with lymphocytic choriomeningitis virus: implications for adoptive immunotherapy of virus carriers. Proc. Natl Acad. Sci. USA.

[31] Berger DP, Homann D, Oldstone MB (2000). Defining parameters for successful immunocytotherapy of persistent viral infection. Virology.

[32] Aubert RD (2011). Antigen-specific CD4 T-cell help rescues exhausted CD8 T cells during chronic viral infection. Proc. Natl Acad. Sci. USA.

[33] Warfield KL (2005). Induction of humoral and CD8+ T cell responses are required for protection against lethal Ebola virus infection. J. Immunol..

[34] Bradfute SB, Warfield KL, Bavari S (2008). Functional CD8+ T cell responses in lethal Ebola virus infection. J. Immunol..

[35] Lukashevich IS (2005). A live attenuated vaccine for Lassa fever made by reassortment of Lassa and Mopeia viruses. J. Virol..

[36] Goicochea MA (2012). Evaluation of Lassa virus vaccine immunogenicity in a CBA/J-ML29 mouse model. Vaccine.

[37] Cartwright HN, Barbeau DJ, McElroy AK (2020). Rift valley fever virus is lethal in different inbred mouse strains independent of sex. Front. Microbiol..

[38] Dodd KA (2014). Rift valley Fever virus encephalitis is associated with an ineffective systemic immune response and activated T cell infiltration into the CNS in an immunocompetent mouse model. PLoS Negl. Trop. Dis..

[39] Bird BH, Albariño CG, Nichol ST (2007). Rift Valley fever virus lacking NSm proteins retains high virulence in vivo and may provide a model of human delayed onset neurologic disease. Virology.

[40] McElroy AK, Albariño CG, Nichol ST (2009). Development of a RVFV ELISA that can distinguish infected from vaccinated animals. Virol. J..

