# Supplementary material for: Immune correlates of protection following Rift Valley fever virus vaccination
Source: NPJ Vaccines. 2022 Oct 28;7:129. doi: 10.1038/s41541-022-00551-4 (PMC9616434; doi:10.1038/s41541-022-00551-4)
Supplement: Supplementary file 1 — Supplemental Material [file 41541_2022_551_MOESM1_ESM.pdf]

Supplemental Figure 1: T Cell Depletion Confirmation

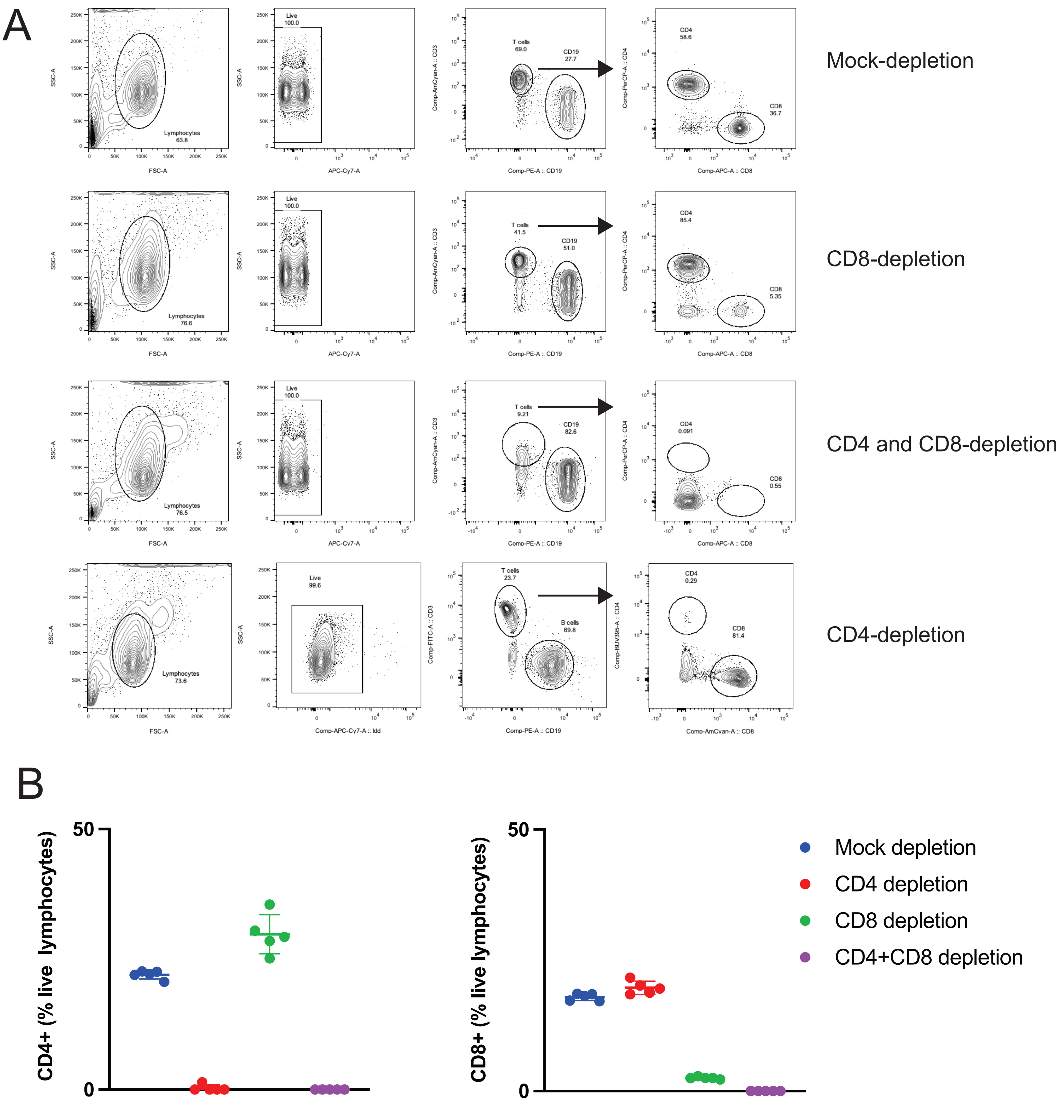

Supplemental Figure 1. Lymphocyte depletion was confirmed by flow cytometry of splenocytes on day of euthanasia. Representative gating strategy is shown for each experimental group (A). The percentages of total live lymphocytes that were CD4+ or CD8+ (B) in mice that underwent mock depletion, CD4+ depletion, CD8+ depletion, or CD4+ and CD8+ depletion are shown.

Supplemental Figure 2: B Cell Depletion Confirmation

A

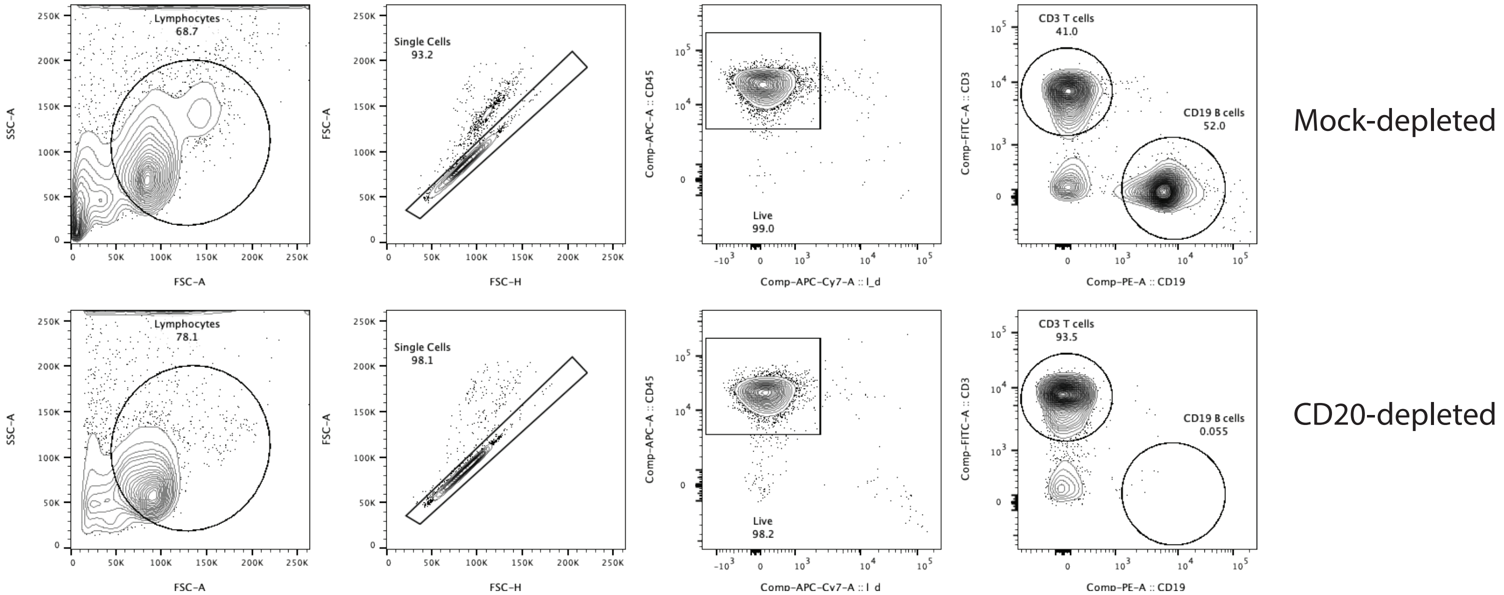

B

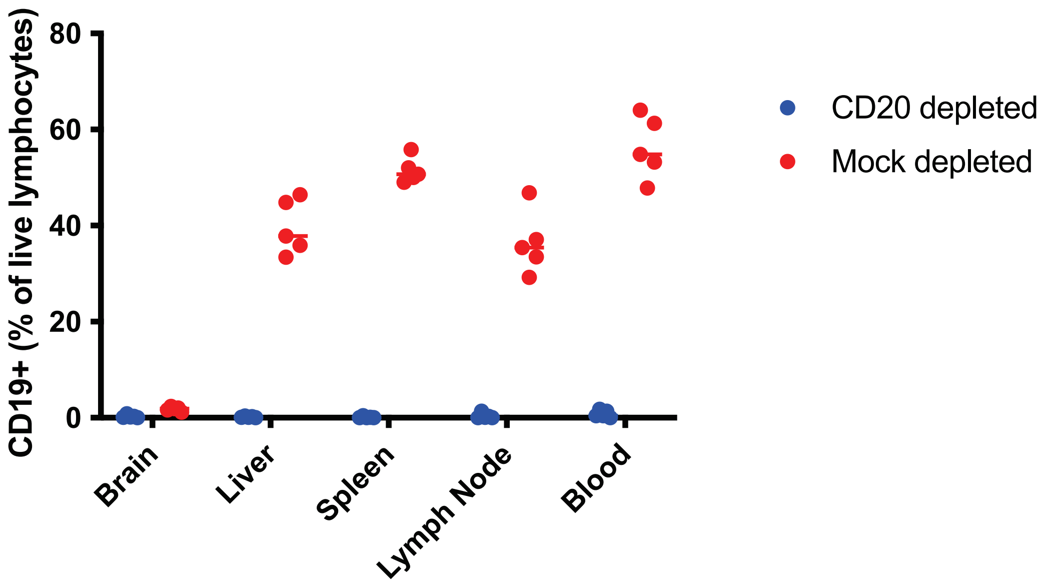

Supplemental Figure 2. B cell depletion was confirmed by flow cytometry of brain, liver, spleen, lymph node, and blood on day 50. Representative gating strategy is shown for mock- and CD20- depleted splenocytes (A).The percentage of total live lymphocytes that were CD19+ was determined in mock depleted and CD20+ depleted mice (B).

Supplemental Figure 3

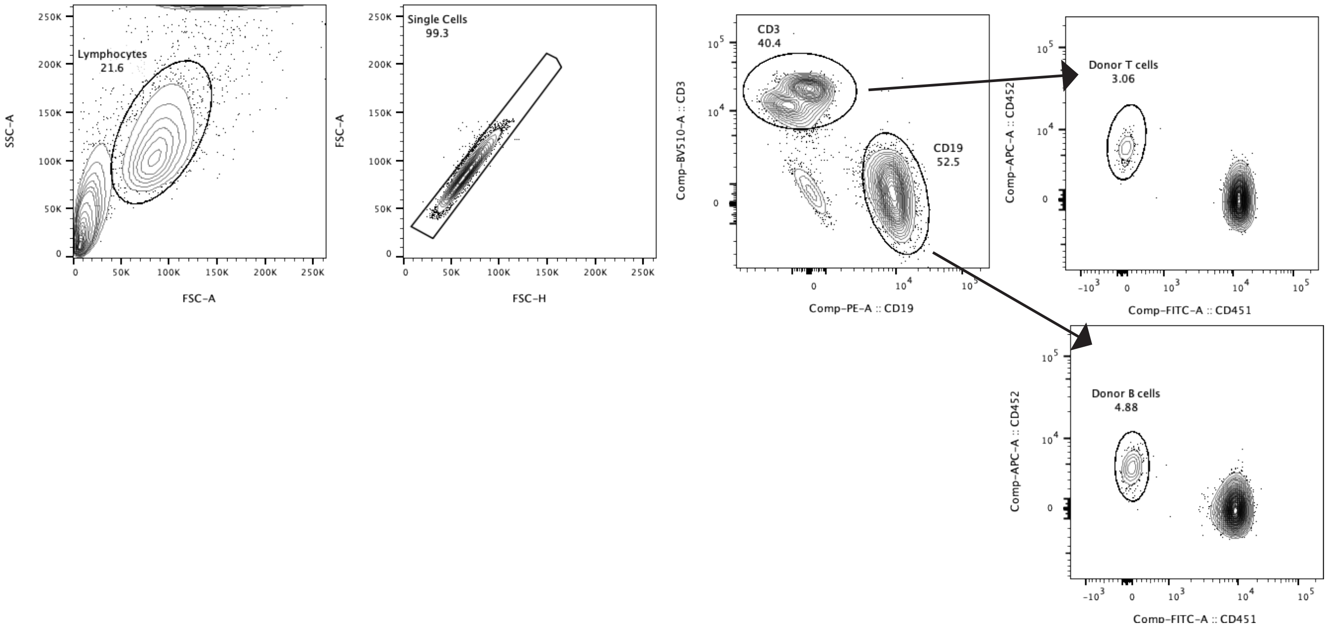

Supplemental Figure 3. Representative gating strategy to distinguish donor from recipient lymphocytes in adoptive transfer experiments.

Supplemental Figure 4

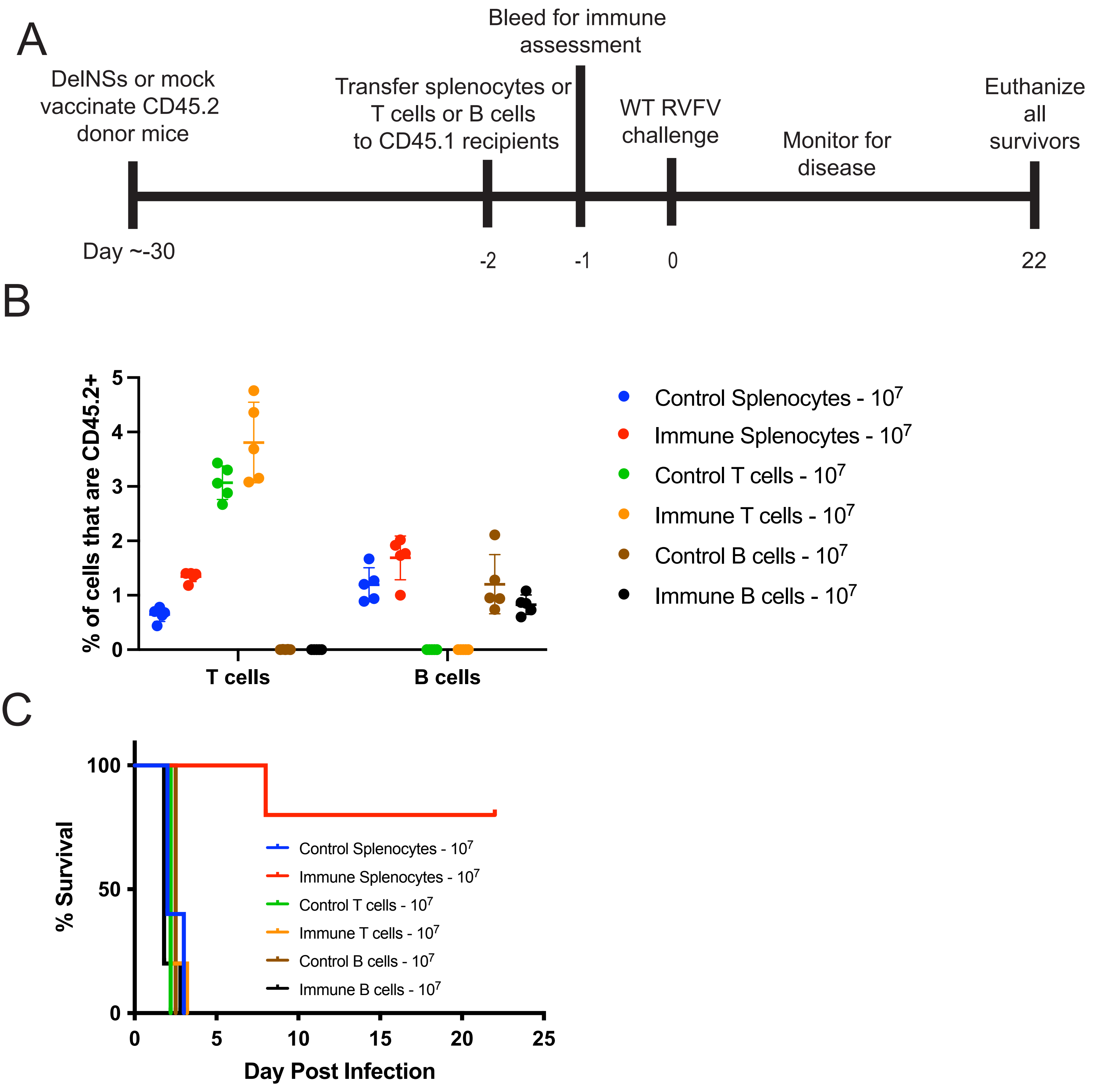

Supplemental Figure 4.Naïve CD45.2 mice (n=5 per experimental group) were vaccinated with 2x105 TCID50 of DelNSsRVFV or mock-vaccinated. 1x107 control or immune splenocytes, T cells or B cells were harvested and transferred to naïve CD45.1 recipient mice prior to WT RVFV challenge with 2x 105 TCID50 WT RVFV (A). The number of donor derived CD45.2 cells present in the blood of recipient animals one day after transfer was determined by flow cytometry (B). Mean and standard deviation are noted. Survival post WT challenge was monitored for 22 days (C).
